# Risk factors for posttraumatic stress reactions among chinese students following exposure to a snowstorm disaster

**DOI:** 10.1186/1471-2458-11-96

**Published:** 2011-02-12

**Authors:** Daxing Wu, Huifang Yin, Shujing Xu, Ying Zhao

**Affiliations:** 1 The Medical Psychological Research Institute, Second Xiangya Hospital of Central South University, Changsha, China

## Abstract

**Background:**

It is important to understand which factors increase the risk of posttraumatic stress disorder (PTSD) in adolescents. Previous studies have shown that the most important risk factors for PTSD include the type, severity, and duration of exposure to the traumatic events.

**Methods:**

A cross-sectional survey was used to investigate the psychological symptoms associated with the aftermath of a snowstorm disaster in the Hunan province of China in January 2008. Students living in Hunan were surveyed at a three**-**month follow-up after the disaster. The questionnaire battery included the Impact of Event Scale-Revised (IES-R, trauma and symptoms associated with PTSD), the Chinese version of the Life Orientation Test-Revised (LOT-R, optimism and pessimism), the Chinese version of the Eysenck Personality Questionnaire (EPQ, neuroticism and extraversion), the Chinese Trait Coping Style Questionnaire (TCSQ, positive and negative coping styles), and a range of questions addressing social demographic characteristics and factors relating to the snowstorm. The survey was administered in school, and 968 students completed and returned the questionnaires.

**Results:**

The results showed that 14.5% of the students had a total IES-R score ≥20. Students with greater school-to-home distances showed higher levels of posttraumatic stress symptoms than students who lived shorter distances from school. Students with emotional support from their teachers reported higher levels of posttraumatic stress symptoms (21.20%) than students without a teacher's emotional support (11.07%). The IES-R total and subscale scores correlated with all variables except extraversion. The binary logistic regression analysis results showed that the teacher's emotional support [odds ratio (OR) = 1.72, 95% confidence interval (CI) = 1.13-2.62], school-to-home distance (OR = 1.01, 95% CI = 1.00-1.01), negative coping (OR = 1.05; 95% CI = 1.02-1.08), and neuroticism (OR = 1.04, 95% CI = 1.02-1.06) were risk factors that predicted PTSD frequency and severity (percentage correct = 85.5%).

**Conclusions:**

The risk factors that significantly impacted the onset of posttraumatic stress reactions in students living in Hunan, China following a snowstorm disaster were the school-to-home distance, negative coping, neuroticism, and teacher's emotional support.

## Background

It is important to understand which factors increase the risk of posttraumatic stress disorder (PTSD) in youth because traumatic events are relatively common during childhood and adolescence. Previous studies have shown that children and adolescents who are exposed to traumatic experiences during disasters may suffer from high levels of posttraumatic stress (Yule et al. 2000; Caffo & Belaise, 2003)[1,2]. The rate of PTSD varies with the type (e.g., tsunami) and severity of the event and the duration of exposure, which was reported to be one of the most important risk factors for PTSD (Yule et al. 2000; Donnelly & Amaya-Jackson, 2002; Piyasil et al. 2007)[3,4].

Studies showed that a simple risk score model could be used to predict PTSD among flood victims (Huang et al. 2010; Mason et al. 2010)[5,6]. Other research suggested that exposure to the World Trade Center site was associated with an elevated PTSD risk as well as functional impairment (Berninger et al. 2010) [7]. Combat exposures, specifically the threat of death, serious injury, and witnessing injury or death, are known to be significant risk factors for post-deployment PTSD among male Marines (Phillips et al. 2010) [8]. Another research has shown that having more social support may lead to less PTSD following exposure to natural disasters (e.g., floods, hurricanes) (Feng et al. 2007; Kaniasty & Norris, 2008)[9,10]. Growing evidence indicates that the coping strategies that individuals utilize are a key predictor of distress following trauma (Ruggiero et al. 2009; Littleton et al. 2010)[11,12].

There are a limited number of studies that have examined PTSD among students in Mainland China. One study reported that 17 months after experiencing an earthquake, the prevalence of PTSD in Chinese students was 9.4%. Predictors of PTSD were gender (female), sad levels, fear of loss of life, and severity of injury (Zhao et al., 2001)[13].

According to Chinese traditional culture, white snow is lucky. An ancient Chinese expression states, "A snow year, a rich year," and this is a widely held cultural belief and expectancy. The first heavy snow is considered a positive event. However, if multiple snowstorms occur within a month, this good event becomes a negative natural disaster that is traumatic and stressful. Beginning on January 10, 2008, a heavy snowstorm began in China, and given the destructive nature of this particular storm, it could be construed as a risk factor for PTSD. In the midst of Chinese New Year, heavy snowfall, sleet, and freezing temperatures blanketed much of central, eastern, and southern China. Snowstorms are rare in southern China, and communities in the region lacked the necessary experience and equipment to cope with this storm, which was China's worst in 50 years. The region's infrastructure was paralyzed, including transportation via road, railway, and air. The storm was directly responsible for at least 151.65 billion Chinese yuan (22.7 billion U.S. dollars) in economic damage. To date, the adverse psychological impact of this disaster on students has not been studied. The psychological effects of a disaster of this magnitude have never been reported in southern China. In particular, we know little about the natural history of PTSD in the pubertal students, who were directly or indirectly exposed to the snowstorm disaster.

After the snowstorm began, the Ethnic Senior School of Baojing in Hunan was particularly hard hit, and the facilities were limited. For example, the classrooms and dormitories were without heat. Food, water, and power supplies were cut off, and the icy roads prohibited students from returning home by bus. On January 25, all urban students were able to return home. The rural students, however, were forcibly kept at school. On January 29, the school leaders decided that eight teachers would personally escort students living in partially remote, rural areas home by walking. The mean walking distance from the school to the students' homes was 50 km, and the distances ranged from 10 km to 120 km. The mean walking time was 9 h and ranged from 4 to 48 h. The students walked home through horrible conditions over very dangerous, mountainous terrain. All the students who lived in extremely remote areas had to walk home through the storm without an escort and without food and water. They suffered from life-threatening cold and frostbite. This was atypical traumatic stress for this sample. According to student self-reports after the event, the students experienced very difficult circumstances.

Baojing has a population of 250,000 and is located in the Xiangxi Tujia and Miao Autonomous Prefecture. It is a mountainous area in western Hunan province with elevations of 800 to 1000 m. In 2000, the Chinese Government defines poverty as a net per capita income of 625 to 865 yuan (75 to 104 U.S. dollars) a year. Of the 592 poverty-stricken counties named on the government's priority poverty relief list from 2001 to 2010, 100% were situated in the central and western regions of the country. Of these regions, 17% were old revolutionary base areas, 45% were in the ethnic minority areas, and 7.3% were located in border areas. According to the government's definition, Baojing County is one of the 592 poverty-stricken counties in China, since it has an average per capita income of 625 yuan (75 U.S. dollars) a year [14].

In this study, we examined the occurrence and three-month course of psychopathological reactions to the snowstorm disaster in a sample of students in China's poorest area. This study had three objectives. First, we hypothesized that the extent of disaster exposure and the severity of posttraumatic stress symptoms would be associated with the student's residence, school-to-home distance, and time to walk home. Second, we examined the impact of the teacher's emotional support while walking the student home. Third, we evaluated the effects of negative coping, pessimism, and neuroticism on the frequency and severity of posttraumatic stress symptoms during the three months following the snowstorm disaster.

## Methods

### Population and Data Sources

A cross-sectional survey was used to investigate the psychological symptoms associated with the aftermath of the snowstorm disaster three months following the event. The students perceived a type of threatening experience while walking home in the snowstorm disaster. This involved the threat of death (e.g., being thrown off the steep cliffs) or serious injuries of themselves or their classmates in the hazardous conditions (e.g., freezing and falling injuries, and fear of ice blocks falling on them off cliffs). Many students suffered hunger or thirst, and these students reported more experiences of fear. Before the traumatic event occurred, all of the students attended class, and no other stressful and traumatic events had occurred at school.

According to the student's self-reports, this traumatic event and the level of exposure met two of the DSM-IV conditions for the diagnostic Criterion A for PTSD [15]. Prior to participation, students received informed consent forms detailing the aims of the study. Only students who signed and returned the informed consent form to the project coordinator were permitted to participate. The study was conducted three months after the students walked home through the winter storm. A total of 1024 students were sent questionnaires to assess their posttraumatic stress reactions, and 968 returned the initial questionnaires (94.5%). The study was approved by the Second Xiangya Hospital Ethics Committees. Detailed information about the study was provided verbally to the students. All students gave written informed consent.

The variables included the student's age, gender, ethnic group, and residence, the teacher's emotional support, the walking time to home, and the school-to-home distance. The mean age of the students was 18.56 years (standard deviation (SD) = 0.85). The 968 students included 428 males (44.2%) and 540 females (55.8%). In terms of the area of residence, 640 students resided in rural areas (66.1%), and 328 students resided in urban areas (33.9%). The ethnicity of the students consisted of 726 Tujia (75.0%), 179 Miao (18.5%), 56 Han (5.8%), and 7 students of other ethnicities (0.7%). The students walked home in one of the two ways: 325 students walked home with a teacher (33.6%), and 643 students walked home without a teacher (66.4%). In terms of the walking time home, 496 students (51.2%) spent less than 2 h, 172 students (17.8%) spent from 2 to 5 h, and 300 students (31.0%) spent over 5 h. With respect to the school-to-home distance, 407 students (42.0%) lived less than 10 km from the school, 139 students (14.4%) lived from 10 to 20 km from school, and 422 students (43.6%) lived more than 20 km away from school.

### Measures

The students completed the following scales. The Impact of Event Scale-Revised (IES-R; Weiss et al., 1997; Creamer et al., 2003; Wu et al., 2003; Elhai et al., 2005; Huang et al., 2006)[16-20] was used to measure PTSD symptomatology. The IES-R is a commonly used, psychometrically sound self-report questionnaire for determining PTSD symptomatology following a trauma. The IES-R consists of 22 items, each scored from 0 (no problems) to 4 (frequent problems), and the total score ranges from 0 to 88. The mean IES-R score for PTSD is 20, and a score of ≥20 on the IES-R is used to estimate the prevalence of PTSD symptoms, with higher IES-R scores indicating more symptoms (Feinstein et al., 2002; Hawryluck et al., 2004)[21,22]. The IES-R has three subscales: avoidance (8 questions), intrusion (8 questions), and hyperarousal (6 questions). In our study, the reliability coefficients were: Cronbach's α = 0.90, standardized item α = 0.91, and Guttman Split-half = 0.85. The item-total correlations were significant (r = 0.44-0.68, all *p *< 0.001). Pearson correlations between the total IES-R score and the three subscale scores were calculated. The correlation coefficients for the avoidance subscale score (r = 0.87), intrusion subscale score (r = 0.91), and hyperarousal subscale score (r = 0.84) were significant (all *p *< 0.001). The subscale correlations were 0.71 for avoidance with intrusion, 0.59 for avoidance with hyperarousal, and 0.65 for intrusion with hyperarousal (all *p <*0.001). The validity analysis showed that the intra-class coefficient of the IES-R total score was 0.90.

The Chinese version of the Life Orientation Test-Revised (LOT-R; Scheier et al., 1994; Lai et al., 2000)[23,24] was used to measure optimism and pessimism. For the sample, Cronbach's α for the optimism and pessimism scales were 0.37 and 0.38, respectively. The subscale negative correlations were 0.202 for optimism with pessimism (*p *< 0.001).

The Chinese version of the Eysenck Personality Questionnaire (EPQ; Gong et al., 1984)[25] was used to assess the neuroticism (N) and extraversion (E) personality dimensions. For our sample, Cronbach's α for the neuroticism and extraversion scales were 0.85 and 0.69, respectively. The negative correlation between the neuroticism with extraversion subscales was 0.217 (*p *< 0.001).

The Chinese Trait Coping Style Questionnaire (TCSQ; Jiang et al. 1999) [26] was used to measure positive and negative coping styles. For this sample, Cronbach's α for the positive and negative coping style scales were 0.71 and 0.72, respectively. The negative correlation between the positive and negative coping style subscales was 0.269 (*p *< 0.001).

### Statistical Analysis

To examine differences in demographic, psychological, and winter storm-related variables, we conducted χ2 tests, t-tests, F-tests and Pearson correlations. Relationships between demographic, psychological, and winter storm-related variables were examined using binary logistic regression analysis.

## Results

Table 1 shows the means and standard deviations of the IES-R subscale scores. Additionally, it displays the percentages of students who scored ≥20 on the IES-R criteria for posttraumatic stress symptoms and the scores for each demographic group. The results showed that 14.5% of the students had a total IES-R score ≥20. The teacher's emotional support and the school-to-home distance were significantly associated with the prevalence of posttraumatic stress reactions in the students during the three months following the frozen snowstorm disaster. Students with longer school-to-home distance and longer walking time to go home showed higher levels of posttraumatic stress symptoms compared to students with shorter distances and walking times. Students with teachers (21.2%) reported higher levels of posttraumatic stress symptoms compared to those who walked home without teachers (11.07%). Since the PTSD effect of teacher emotional support was influenced by the school-to-home distance variable, an analysis of covariance was performed, and the results showed that the teacher's emotional support (F_1,967 _= 6.765, *p *< 0.01) and the school-to-home distance (F_1,967 _= 9.488, *p *< 0.01) variables were significantly associated with the prevalence of posttraumatic stress reactions.

**Table 1:** IES-R subscale scores and the prevalence of posttraumatic stress reactions according to the student demographics.

Characteristic		IES-R						χ2
			
		N	Avoidance	Intrusions	Hyperarousal	Total score	% of ≥20	
Total		968	2.64 ± 3.43	4.10 ± 4.04	2.67 ± 3.23	9.89 ± 9.76	14.5	
								
Gender	Male	428	2.82 ± 3.71	4.31 ± 4.35	2.49 ± 3.14	10.01 ± 10.41	14.3	0.03
	Female	540	2.49 ± 3.18	3.93 ± 3.78	2.81 ± 3.30	9.80 ± 9.23	14.6	
	*t*		1.45	1.42	1.52	0.34		
								
Age (y)	≤18	499	2.58 ± 3.37	3.99 ± 4.14	2.66 ± 3.35	9.74 ± 10.10	14.4	0.00
	>18	469	2.70 ± 3.49	4.21 ± 3.94	2.68 ± 3.10	10.05 ± 9.40	14.5	
	*t*	0.51	0.84	0.10	0.48			
								
Ethnicity	① Tujia	726	2.54 ± 3.33	3.89 ± 3.91	2.61 ± 3.17	9.53 ± 9.55	13.5	5.65
	② Miao	179	3.07 ± 3.85	5.15 ± 4.67	3.08 ± 3.52	11.79 ± 11.00	19.6	
	③ Han	56	2.68 ± 3.39	3.66 ± 3.14	2.14 ± 2.90	8.89 ± 7.84	12.5	
	④ Other	7	1.29 ± 1.50	2.43 ± 2.15	2.43 ± 3.91	6.57 ± 6.11	0.0	
	F		1.53	5.40**	1.55	3.08*		
	LSD			② > ①** ② >③*		② > ①**		
Teacher emotional support								
yes	325		3.26 ± 3.59	5.18 ± 4.25	3.03 ± 3.31	12.01 ± 10.08	21.2	18.12**
no	643		2.32 ± 3.29	3.55 ± 3.82	2.48 ± 3.17	8.82 ± 9.42	11.0	
	*t*		3.97**	5.86**	2.52*	4.76**		
								
Walking time to home^a^	① 0~2	496	2.11 ± 2.91	3.31 ± 3.47	2.29 ± 2.90	8.21 ± 8.33	9.9	17.62**
	② 2~5	172	2.92 ± 4.05	4.37 ± 4.35	2.95 ± 3.34	10.74 ± 10.84	18.0	
	③ >5	300	3.34 ± 3.69	5.24 ± 4.44	3.13 ± 3.59	12.18 ± 10.76	20.0	
	F		13.09**	22.80**	7.27**	16.75**		
	LSD		③ > ①** ② > ①**	③ > ①** ② > ①** ③ >②*	③ > ①** ②> ①*	③ > ①** ② > ①**		
School-to-home distance^b^	① 0-10	407	2.10 ± 3.02	3.26 ± 3.56	2.18 ± 2.91	8.04 ± 8.67	8.8	21.46**
	② 10-20	139	2.70 ± 3.52	3.96 ± 3.89	2.75 ± 3.35	9.81 ± 9.32	13.7	
	③ >20	422	3.14 ± 3.69	4.95 ± 4.35	3.11 ± 3.42	11.70 ± 10.54	20.1	
	F		9.73**	18.81**	8.64**	15.03**		
	LSD		③ > ①**	③ > ①** ③> ②*	③ > ①**	③ > ①** ③> ②*		

*Note*. Data are represented as the mean ± SD. a = hours; b = kilometers; * *p *< 0.05, ** *p *< 0.01.

Table 2 shows the mean scores, standard deviations, and correlations between all the measures. The IES-R total and subscale scores correlated positively with the negative coping scores, LOT-R pessimism, and EPQ neuroticism, and they correlated negatively with positive coping and the LOT-R pessimism scores except for EPQ extraversion.

**Table 2:** Mean scores and correlations between the IES-R total score and psychological variables.

	Mean ± SD	IES-R total score	Avoidance	Intrusion	Hyperarousal
Positive coping	32.97 ± 6.69	-0.139**	-0.097**	-0.090**	-0.179**
Negative coping	26.86 ± 7.89	0.306**	0.217**	0.231**	0.353**
LOT-R optimism	11.43 ± 2.21	-0.130**	-0.081*	-0.099**	-0.164**
LOT-R pessimism	6.36 ± 2.37	0.162**	0.146**	0.110**	0.164**
EPQ extraversion	52.50 ± 21.49	-0.050	-0.051	-0.038	-0.048
EPQ neuroticism	46.68 ± 12.20	0.312**	0.180**	0.260**	0.371**

* p < 0.05, ** p < 0.01.

Table 3 shows the binary logistic regression analysis results for the PTSD dependent variables (Wald = 4.89, df = 1, p < 0.05; percentage correct = 85.5%). The analysis indicated that teacher's emotional support (odds ratio (OR) = 1.72, 95% confidence interval (CI) = 1.13-2.62), school-to-home distance (OR = 1.01, 95% CI = 1.00-1.01), negative coping (OR = 1.05; 95% CI = 1.02-1.08), and EPQ neuroticism (OR = 1.04, 95% CI = 1.02-1.06) were significant predictors of PTSD reactions. None of the other variables were associated with posttraumatic stress symptoms (all p > 0.05).

**Table 3:** Binary logistic regression analysis of PTSD risk factors.

Predictor variable	B	**S.E**.	OR	95% CI	Wald	p
Constant	-5.19	2.35	0.01		4.89	0.027
Gender^a^	-0.21	0.21	0.82	0.55-1.22	0.99	0.319
Age	-0.05	0.11	0.96	0.77-1.19	0.17	0.680
Ethnicity^b^	0.05	0.20	1.06	0.72-1.55	0.08	0.783
Teacher emotional support^c^	0.54	0.22	1.72	1.13-2.62	6.37	0.012
School-to-home distance	0.01	0.00	1.01	1.00-1.01	4.80	0.029
Positive coping	-0.01	0.02	0.99	0.96-1.03	0.26	0.610
Negative coping	0.05	0.02	1.05	1.02-1.08	10.06	0.002
LOT-R optimism	-0.02	0.05	0.98	0.89-1.07	0.27	0.602
LOT-R pessimism	0.07	0.04	1.07	0.99-1.16	2.68	0.102
EPQ extraversion	0.02	0.01	1.02	0.10-1.04	2.83	0.093
EPQ neuroticism	0.04	0.01	1.04	1.02-1.06	12.37	0.001

*Note*. Dependent variable: PTSD; OR = Odds ratio; CI = Confidence interval; ^a ^0 = male, 1 = female; ^b ^0 = Han 1 = Tujia 2 = Miao 3 = other; ^c ^0 = no 1 = yes.

## Discussion

The results showed that students with longer school-to-home distances showed higher levels of posttraumatic stress symptoms than students who lived shorter distances from school. Students with emotional support from their teachers reported higher levels of posttraumatic stress symptoms than students without a teacher's emotional support. The IES-R total and subscale scores correlated with all variables except EPQ extraversion. The binary logistic regression analysis results showed that the teacher's emotional support, school-to-home distance, negative coping, and neuroticism were risk factors that predicted PTSD frequency and severity (percentage correct = 85.5%).

The percentage of all students with total IES-R scores ≥20 was 14.5%, which is similar to the results of Giaconia et al. (1995)[27], who showed that more than 40% of adolescents experienced at least one DSM-III-R trauma symptom by the age of 18, and 14.5% of these affected youths developed PTSD (6.3% of the total sample). Piyasil et al. (2007)[4] showed that the prevalence of PTSD in affected students after the 2004 Thailand tsunami was 57.3% at 6 weeks, 46.1% at 6 months, 31.6% at 1 year, 10.4% at 1.5 years, and 7.6% at 2 years. Of the 176 students who were directly affected, 48 (27.3%) suffered from PTSD. Meanwhile, of the 1314 students who were not directly affected by the tsunami, but who were among affected friends and relatives, 42 students (3.1%) suffered from PTSD. In comparison to these results, our data indicated a lower prevalence of PTSD in affected Chinese students after the snowstorm. These results suggested that the posttraumatic stress severity of exposure to the snowstorm was lower than the posttraumatic stress severity of exposure to the tsunami.

In our present study, students with longer school-to-home distances and walking time to return home showed higher levels of posttraumatic stress symptoms compared to students with shorter distances and walking time. The findings suggested that the nature of the exposure to the traumatic event appeared to be the most important risk factors for PTSD.

Kaniasty & Norris (2008) and Vigil & Geary (2008)[10,28] showed that exposure to increased levels of community-provided support may have unintended consequences on the psychological health of adolescents experiencing trauma. Moore et al. (2010)[29] demonstrated that lower levels of classmate support and more negative life events after Hurricane Katrina were uniquely related to PTSD symptoms in a group of children 33 months after the hurricane. Our findings did not indicate that teachers' support had a salutary effect on students during their exposure to the winter storm. Students with a teacher's emotional support reported higher levels of posttraumatic stress symptoms (21.2%) than students who walked home without teachers (11.07%). Analysis of the covariance results showed that the teacher emotional support variable was significantly associated with the prevalence of posttraumatic stress reactions. In the binary logistic regression analysis, the teacher's emotional support was entered and made a significant contribution to the explained PTSD variance. This is probably due to the fact that a higher level of emotional support from the teacher would intensify the PTSD susceptibility, and students showed more posttraumatic stress reactions when they had a longer exposure to the winter storm. It is also possible, that students who were perceived to be the most psychologically vulnerable were more likely to be given support. Whether the result is due to a sort of secondary traumatization should a focus of further research.

Hatcher et al (2009)[30] studied spinal cord injury patients and observed that negative cognitions of self and neuroticism were potential risk factors for the development of PTSD. Likewise, our results demonstrated that negative coping and neuroticism correlated positively with the IES-R total and subscale scores. The binary logistic regression analysis results showed that negative coping (OR = 1.05) and neuroticism (OR = 1.04) were risk factors that predicted the frequency and severity of posttraumatic stress reactions (percentage correct = 85.5%).

The limitations of the current study include the absence of baseline data prior to the winter storm's onset and data from one month after the storm. Furthermore, the relatively short follow-up period is a limitation. The lack of structured interviews in assessing PTSD precludes us from determining whether the self-reported symptoms have clinical significance. Although our sample was unique, the absence of a control group is another limitation of the study. Finally, a major limitation of this study is related to the lack of data on the role of mediating factors. Since these variables were not assessed, this limits the ability to study how traumatic events impact the psychopathological aspects of children and adolescents.

## Conclusions

School-to-home distance, negative coping, neuroticism, and teacher's emotional support act as risk factors that significantly influenced the onset of posttraumatic stress reactions in students following a snowstorm disaster in China's poorest area. The results of this study have important implications for the screening, prevention, and treatment of PTSD symptoms in adolescents long after exposure to a disaster.

## Competing interests

The authors declare that they have no competing interests.

## Authors' contributions

DW conceptualized and designed the study. DW, HY, SX, and YZ collected and analyzed the data. DW supervised the study, further analyzed the data, and wrote the final manuscript. All authors read and approved the paper.

## Pre-publication history

The pre-publication history for this paper can be accessed here:

http://www.biomedcentral.com/1471-2458/11/96/prepub
